# Effect of a 6-Week Physical Education Intervention on Motor Competence in Pre-School Children with Developmental Coordination Disorder

**DOI:** 10.3390/jcm10091936

**Published:** 2021-04-30

**Authors:** Rubén Navarro-Patón, Juan Luis Martín-Ayala, Mariacarla Martí González, Alba Hernández, Marcos Mecías-Calvo

**Affiliations:** 1 Facultad de Formación del Profesorado, Universidade de Santiago de Compostela, 27001 Lugo, Spain; ruben.navarro.paton@usc.es; 2 Facultad de Ciencias de la Salud, Universidad Europea del Atlántico, 39011 Santander, Spain; juan.martin@uneatlantico.es (J.L.M.-A.); mariacarla.marti@uneatlantico.es (M.M.G.); alba.hernandez@uneatlantico.es (A.H.); 3 Departamento de Educación, Universidad Internacional Iberoamericana, Campeche 24560, Mexico; 4 Centro de Investigación y Tecnología Industrial de Cantabria (CITICAN), 39011 Santander, Spain

**Keywords:** childhood, schoolchildren, Movement Assessment Battery for Children-2 (MABC-2), motor competence intervention, disorders in motor development

## Abstract

The objective of this research was to analyze the impact of an intervention program performed by a specialist in physical education (PE) to contribute to the development of motor competence (MC) in pre-school children with motor development problems. The sample consisted of 28 children (12 from the intervention group and 16 from the control group) aged between 4.1 and 5.9 years (mean = 4.71 ± 0.54) who were in the fifth and sixth grades of pre-school education in two schools from Lugo, Spain. The Movement Assessment Battery for Children-2 (MABC-2) was used for data collection. The data revealed that, in the pre- and post-test intervention groups, there are statistically significant differences in manual dexterity (*p* < 0.001; *d =* 2.63), aiming and catching (*p* < 0.002; *d =* 1.13), balance (*p* < 0.001; *d =* 1.68), total test score (*p* < 0.001; *d =* 3.30) and total percentile score (*p* < 0.001; *d =* 1.88). Between the control and intervention post-test groups, significant differences were found in manual dexterity (*p =* 0.015; η^2^ = 0.22), aiming and catching (*p =* 0.003; η^2^ = 0.32), balance (*p* = 0.050; η^2^ = 0.15), total test score (*p* < 0.001; η^2^ = 0.47) and total percentile score (*p* < 0.001; η^2^ = 0.48). Based on the results obtained, a specific MC program implemented by a PE specialist contributed to the improvement of manual dexterity, aiming and catching and balance, as well as a better percentile in the general MC of pre-school children diagnosed with motor skill problems.

## 1. Introduction

Childhood is a particularly important period for the development of motor competence (MC) in pre-school children and the foundation for future development, and its delay can have long-lasting negative effects [1]. These effects include an increased risk of being overweight [2], difficulties in cognitive functions [3] and problems in physical self-concept [4], as well as less participation in physical and sports activities [5,6]. The acquisition of an adequate MC will be determined by the natural maturation and development of children, as well as by the continuous interaction with the environment, both social and physical [7,8]. Motor skills are acquired, fundamentally, during childhood, through movement and body control habits and the acquisition of autonomy in the development of habitual activities [9,10]. Thus, it will collaborate with the subsequent development of more complex and specialized motor skills [5,11,12] and possible promotion of an active lifestyle [13,14,15]. 

The presence of a poor ability to perform and learn age-appropriate motor skills is known as developmental coordination disorder (DCD) [16], as long as there is no medical condition or neurological dysfunction causing it. Despite knowing the importance of MC in physical activity and health habits, studies such as that by Roth et al. [17] reveal that pre-school children do not develop this competence adequately, which is why a large number of children have motor delays during this stage. 

In this sense, DCD is the most common disorder affecting school-age children, ranging between 5 and 19% of schoolchildren in developed countries [18,19,20,21]. In our country of Spain, almost one in five schoolchildren have a probable DCD or are at risk of having movement problems [22]. These children present motor difficulties that cause poor learning and execution of activities related to fine and gross motor skills [23] and balance [24], as well as everyday activities [25] such as outdoor games and activities, or social participation, and especially performance in school [26,27,28]. 

Monitoring children’s motor aptitude is a preventive attitude that should be implemented in schools because early detection of disorders in motor development could enable discovering the limitations and needs [29]. Based on these limitations and needs, an appropriate group intervention can be programmed through specific programs in schools [29,30]. Therefore, it is necessary to design and carry out programs for improving MC in children [22], although its evaluation in children with motor difficulties is problematic [31]. There are several standardized tests to assess MC and detect DCD in pre-school children, including the Movement Assessment Battery for Children, Second Edition (MABC-2). In fact, the European Academy of Childhood Disability recommends using the MABC-2 to assess motor coordination performance and detect pre-school children with percentiles substantially lower than expected for their chronological age [32].

As can be seen, only with adequate intervention can children with DCD improve their MC to normal levels [33]. In this sense, due to the large amount of time that children spend in schools, it becomes the best place for the development of physical activity programs [22], specifically in physical education (PE) classes, as these play a vital role in children’s motor development [34,35,36], among other factors.

Scientific evidence indicates that interventions are effective in the short term to improve MC, as well as performance in cognitive, emotional and other psychological aspects in children with DCD [37,38,39]. Furthermore, this evidence indicates that difficulties in MC can continue into adolescence and into adulthood if these specific interventions are not applied [32]. Interventions in children with DCD are based on individual therapeutic interventions [40]; however, group interventions are effective for these children and should be considered a feasible, efficient and cheaper treatment option than individual ones [30]. There are few group interventions, and they report contradictory results. Some report that there are no differences after the intervention [30,41], and others report positive results with this type of intervention [42]. Furthermore, these interventions, for the most part, last between 8 and 10 weeks [30,41] and were not tested with interventions of shorter duration. Therefore, more interventions are needed to investigate the immediate effects they create on MC and motor performance in children with DCD [39]. In our country, no previous studies have been carried out on the influence of a specific intervention on MC in children with DCD, so the objective of this study was to determine whether a specific 6-week intervention on MC produces immediate effects in the skills of manual dexterity, aiming and catching, and balance, in children who present problems of development in MC.

## 2. Materials and Methods

### 2.1. Study Design

To carry out this research, a quasi-experimental design was carried out with pre- and post-test measures with a control group [43]. The variables of the Movement Evaluation Battery for Children-2 (MABC-2) were the dependent variables, comparing them according to group (Control vs. Intervention).

### 2.2. Participants

Two schools in the city of Lugo, Galicia (Spain) were invited to participate in the research. After they approved to participate, an MC study was carried out in their 4- to 6-year-old students. Once the motor tests of the MABC-2 Battery had been carried out, those pre-school children below the 5th percentile were selected.

### 2.3. Measurements

MABC-2 [31] was used to determine the MC of pre-school children in Spain. It is a valid and reliable test to identify changes in motor competence in pre-school children [31,44,45,46] with very high inter-rater reliability [47]. This battery enabled knowing the evolution in the MC of pre-school children through the analysis of three specific skills (manual dexterity, aiming and catching and balancing; registered through scalar scores using the equivalence tables provided by the MABC-2 battery), developing eight standardized tests for each skill (Table 1).

The total score was also interpreted in terms of a “traffic light” system designating three zones (i.e., green: performance within the normal range (percentile above 16th); amber: performance within the “risk zone” where the child needs careful monitoring (6–15 percentile); red: motor competence problems (percentile less than 5)).

### 2.4. Procedures

To find out the participation from the schools, teachers and parents/legal guardians of pre-school children, an information sheet was delivered to them explaining the purpose, design and procedure of the study (data recording, analysis techniques and their subsequent use) and the latter, an informed consent form (declaration of confidentiality, voluntary participation and the possibility of withdrawing the child from the study at any time), progressively as approval was given.

Once accepted by all parties, the MABC-2 assessments and sociodemographic data (sex and age) of the participants began to be recorded. In this sense, the evaluation was always carried out under the same conditions for each child: individual, with comfortable clothes, at school and with standardized material. For this, the evaluators always followed the same application methodology in all schools, following the instructions of the battery. Before the assessments, the students were allowed to take a test where the examiners could correct some aspects of the test. Once the test started, the examiners did not apply any type of correction.

Once the students of the two centers were evaluated, they were randomized by natural groups (belonging to the same group classroom and school center) to facilitate the development of the program. 

Within the CG, non-specialist PE teachers continued with their usual planning and developing the PE curriculum for pre-school education in Spain (that is, the body and their own body image, play and movement, daily activity and personal care and health) [48], without knowing the intervention that was carried out with the IG or that they were participating in it. It should be noted that neither the duration, the frequency, nor the exact content to be worked on in the CG were recorded. In the IG, the intervention replaced PE classes and were designed and taught in the sports facilities of each school by a graduate in PE with more than 20 years of experience in educating children and more than 10 years in training PE teachers in pre-school and primary education. This intervention consisted of one session of 40 min per week for 6 weeks (i.e., 270 min). Each session began with a warm-up or welcome activity (5 min), three or four tasks related to the skill to be developed (manual dexterity, aiming and catching or balance; 30 min) and a cool-down or goodbye activity (5 min). The structuring and distribution of the sessions were carried out based on the different objectives to be developed according to each skill (Table 2).

The day after the end of the 6 weeks of intervention, the MABC-2 battery was reapplied for both groups (CG and IG).

### 2.5. Ethics

The research was approved by the Ethics Committee of the national EDUCA platform (Code 22019), in accordance with the recommendations of the Declaration of Helsinki.

### 2.6. Statistical Analysis

Firstly, to establish that the groups were equivalent with respect to age and gender, an independent samples *t*-test and a chi-square test were used, respectively. The differences in the pre-test and post-test in all the variables of the MABC-2 battery according to group (CG vs. IG) and gender (boys vs. girls) were evaluated by means of a multivariate analysis of variance (MANOVA). The effect size was calculated using eta squared (η^2^) and the interaction between variables using the Bonferroni statistic to know the statistical significance. Once the intervention process was applied in the PE classes, the *t*-test of related samples was used to evaluate the changes produced in each of the groups (CG vs. IG). Statistical power was expressed by Cohen’s d statistic, with *d* = 0.20 small, *d* = 0.50 moderate and *d* = 0.80 large. SPSS software (SPSS v.25, IBM Corporation, New York, NY, USA) was used for all statistical analyses. The level of significance was set at *p* < 0.05.

## 3. Results

A total of 28 pre-school children with significant motor skill difficulties (percentile equal to or less than 5 obtained in the MABC-2 battery) participated in this study. Six (21.4%) were girls, and 22 (78.6%) were boys aged 4–6 years old (mean = 4.71; SD = 0.54). The distribution of the participants was 16 pre-school children from the CG and 12 pre-school children from the IG, respectively.

### 3.1. Baseline Characteristics

Participants in the CG and the IG were similar at baseline for gender (*p =* 0.595) and age (*p =* 0.203). MABC-2 baseline characteristics are outlined in Table 3.

The results of the multivariate analysis (MANOVA; Table 3) before the intervention, in terms of manual dexterity, indicate that there is no significant main effect in the type of group factor (*p =* 0.907) nor in the gender factor (*p =* 0.907), but in the interaction between both factors (F (1, 24) = 21.256, *p* < 0.001, η^2^ = 0.47], these differences are between the boys and girls of the IG (*p* = 0.010) and the CG (*p* < 0.001), between the CG and IG girls (*p* = 0.014) and between the CG and IG boys (*p* < 0.001).

For the rest of the MABC-2 variables analyzed (aiming and catching, balance, total test score and total percentile score), no significant differences were found in relation to group, gender or the interaction between them (group and gender).

### 3.2. Control Group Outcomes

**Figure 1:**
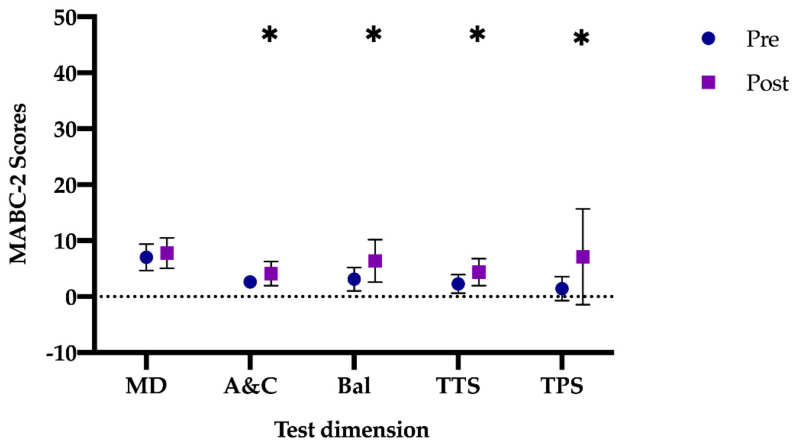
Differences between pre- and post-test in the CG. CG: Control group; MD: manual dexterity; A&C: aiming and catching; Bal: balance; TTS: total test score; TPS: total percentile score. Note: * *p* < 0.001 different between pre- vs. post-test.

After the intervention period, the mean differences in all tests for CG were: manual dexterity (mean difference: −0.75 (95% CI: −2.06–0.56), t (15) = −1.18; *p =* 0.242, *d =* 0.30), aiming and catching (mean difference: −1.50 (95% CI: −2.22–−0.77), t (15) = −4.392; *p* < 0.001, *d =* 1.10), balance (mean difference: −3.25 (95% CI: −4.94–−1.56), t (15) = −4.097; *p* < 0.001, *d =* 1.00), total 8 test scores (mean difference: −2.12 (95% CI: −3.27–−0.97), t (15) = −3.942; *p* < 0.001, *d =* 0.98) and total percentile score (mean difference: −5.71 (95% CI: −9.77–−1.64), t (15) = −2.996; *p =* 0.009, *d =* 0.75). The scores in the different tests show that the differences increased significantly in all the studied variables of the MABC-2, except in manual dexterity (Figure 1).

### 3.3. Intervention Group Outcomes

**Figure 2:**
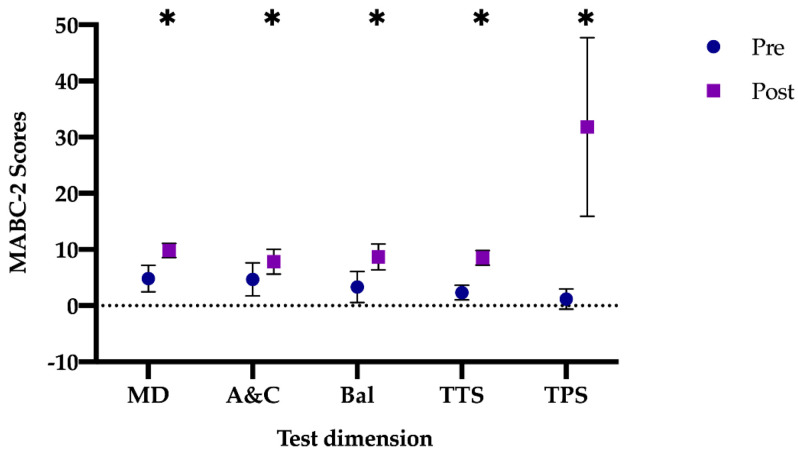
Differences between pre- vs. post-test in the IG. IG: Intervention group; MD: manual dexterity; A&C: aiming and catching; Bal: balance; TTS: total test score; TPS: total percentile score. Note: * *p* < 0.001 different between pre- vs. post-test.

The results obtained regarding the difference between the pre- and post-test in the IG at the overall level (Figure 2) were: manual dexterity (mean difference: −5.00 (95% CI: −6.21–−3.79), t (11) = −9.81; *p* < 0.001, *d =* 2.63), aiming and catching (mean difference: −3.17 (95% CI: −4.93–−1.39), t (11) = −3.931; *p =* 0.002, *d =* 1.13), balance (mean difference: −5.33 (95% CI: −7.34–−3.31), t (11) = −5.825; *p* < 0.001, *d =* 1.68), total test score (mean difference: −6.16 (95% CI: −7.34–−4.99), t (11) = −11.544; *p* < 0.001, *d =* 3.30) and total percentile score (mean difference: −30.65 (95% CI: −40.98–−20.31), t (11) = −6.527; *p* < 0.001, *d =* 1.88). In this case, all scores increased significantly after the application of a specific intervention program in PE classes, with a high effect size and moving to the green level of the MABC-2 battery.

### 3.4. Intervention Group vs. Control Group Outcomes

**Figure 3:**
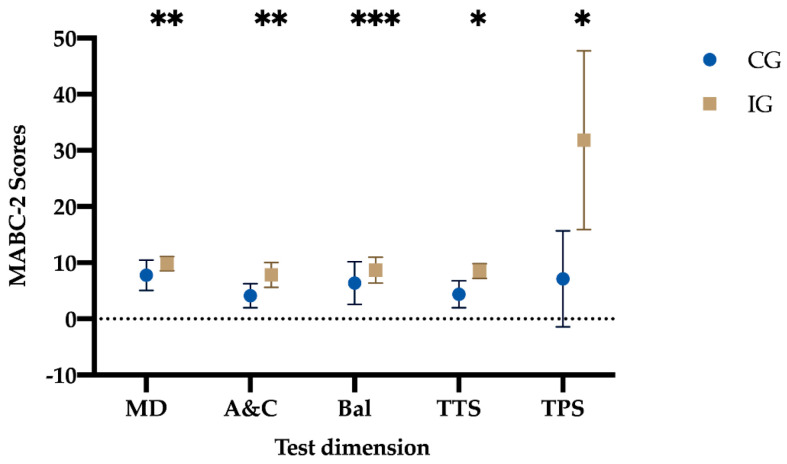
Differences between the CG vs. the IG after the application of the training program. CG: Control group; IG: Intervention group; MD: manual dexterity; A&C: aiming and catching; Bal: balance; TTS: total test score; TPS: total percentile score. Note: * *p* < 0.001 different between the CG vs. the IG; ** *p* < 0.05 different between the CG vs. the IG; *** *p =* 0.05 different between the CG vs. the IG.

The results of the comparisons between the CG and the IG, after applying the training program, were as follows (Table 4, Figure 3). 

The results of the multivariate analysis (MANOVA; Table 4, Figure 3) after the intervention, regarding manual dexterity, indicate that there is a significant main effect in the group factor [F (1, 24) = 6.845, *p =* 0.015, η^2^ = 0.2), greater in the IG. No statistically significant differences were found in the gender factor (*p =* 0.317) nor in the interaction between both factors (*p =* 0.237).

Regarding aiming and catching, the results show that there is a significant main effect in the group factor (F (1, 24) = 11.093, *p =* 0.003, η^2^ = 0.32), with higher scores in GI preschoolers, and in the gender factor (F (1, 24) = 8.847, *p =* 0.007, η^2^ = 0.27), with the highest scores in boys of the IG (*p* < 0.001) compared to boys of the CG. No statistically significant differences were found in the interaction of both factors (*p =* 0.516).

In balance, there is a significant main effect in the group factor (F (1, 24) = 4.185, *p* = 0.05, η^2^ = 0.15), higher in the IG. A significant main effect was also found in the gender factor (F (1, 24) = 4.185, *p* = 0.05, η^2^ = 0.15), higher in girls than in boys.

The results on the total test score indicate that there is a significant main effect in the group factor (F (1, 24) = 21.479, *p* < 0.001, η^2^ = 0.47), greater in the IG. No significant main effect was found in the gender factor (*p* = 0.779) nor in the interaction of both factors (*p* = 0.384).

Finally, with respect to the total percentile score, the results show that there is a significant main effect in the group factor (F (1, 24) = 22.093, *p* < 0.001, η^2^ = 0.48), higher in the IG. No significant main effect was found in the gender factor (*p* = 0.977) nor in the interaction of both factors (*p* = 0.326).

## 4. Discussion

The aim of this study was to find out if a specific intervention in 6-week PE classes can produce improvements in MC in pre-school children with DCD. As expected, the results of this study indicate significant differences between the IG and the CG in all MC measures, as in other studies with similar interventions with healthy [49,50] and older children [40,51,52,53,54]. More importantly, our study confirmed that children with DCD improved in manual dexterity, aiming and catching, balance, total test score and increased percentile after a specific 6-week intervention period in PE classes. These results suggest that a structured program of activities with an emphasis on the motor skills of manual dexterity, aiming and catching and balance can benefit the MC of children with DCD, as has already been demonstrated in previous studies [41,52,55,56]. 

Improvements in the CG were also observed in children with DCD who did not receive the specific operation and who followed their classes, except in manual dexterity, but the differences between the pre- and post-test groups were smaller. These results may be due to the fact that the acquisition of an adequate MC is not only achieved through natural development and maturation, as may have occurred in the control group, but also through its promotion and instruction through specific programs [7,8], as was the case in our study. Even so, the effect size in the CG is medium–high. However, these students remain in the amber-zone percentile of the MABC-2 battery, with a mean of 7.13 (*SD* = 8.55), indicating that these students should be monitored because it can present problems in MC at a global level. 

The main finding of our study is that specific MC-oriented classes, as taught by a specialist, showed a positive effect on all dimensions of the MACBC-2 in the IG. The study of the individual results of the IG revealed that the 12 pre-schoolers improved in the total percentile of the MABC-2 battery to such an extent that they could be classified in a different performance category in this test and change the clinical range to the normal range [57]. As expected, although there were general improvements in the CG, their ranking in the total percentile did not change significantly from the start of the study, as only 4 of the 16 exceeded the 16th percentile of the battery, by which their MC can be considered stable or without significant changes [57]. Our results show that those IG children with lower scores with respect to their total percentile obtained high improvements to the point of falling within a normal CM range (percentile >16), following the line of the results from Farthat et al. [57], which indicates that children with serious problems in MC, after a structured program, benefit more than those who framed in higher percentiles and classified with a normal MC (that is, their possibility of improvement is greater).

With the task-oriented data that the authors have, no further results can be inferred. However, the interventions on this subject normally are of longer duration and of an individual nature. With this type of group intervention in PE classes (the teacher–student ratio is 1/25 in the Spanish educational system), and planned by a PE specialist, good results are achieved with high effect sizes.

Analyzing the skills with the MABC-2 battery with respect to manual dexterity, improvements were made in the IG, which could be because specific tasks were carried out on this skill in the specific intervention. However, in the CG, no improvements were obtained in this subscale, which could be due to the fact that, on the one hand, fine motor skills require longer acquisition time [58], and on the other hand, PE sessions developed by non-specialist teachers do not usually include these types of tasks in their sessions [52]. Regarding aiming and catching, the results show an increase in the score in each of the groups, but they are higher in the IG. These results agree with those of Yu et al. [4] and Woods et al. [59], which indicate that an intervention based on fundamental movement skills improves object control skills in pre-school children with DCD, discarding the idea that these skills are difficult to improve upon [49]. Regarding balance, improvements were also produced after a specific operation on MC, as in previous studies such as that of Yu et al. [4]. The improvements obtained in all the MABC-2 subscales were expected, as, with training through specific manual dexterity, aiming and catching and balance tasks, should produce positive effects on them [52,57,60].

Regarding the limitations of this research, it should be noted that this study uses a quasi-experimental design, and the group assignments were not completely random for logistical reasons. Furthermore, the sample size was small and uneven. On the other hand, the content of the classes followed by the CG was not controlled, so improvements were made based on the content worked during those weeks. Moreover, we cannot know if these improvements would be sustained over time, as a long-term follow-up was not carried out nor if this type of intervention produces an adaptation to the achievement of the objective in pre-school children, so more studies are necessary to evaluate the process.

## 5. Conclusions

The findings of this study showed that a structured 6-week program based on different motor skills (i.e., manual dexterity, aiming and catching), performed in 1 session of 40 min a week, is more effective than a traditional PE program in pre-school children with DCD led by a non-PE teacher, as it improves all these skills and the total percentile in such a way that the pre-school children who participated in the intervention went from being framed in a percentile lower than 5 to one in which they can be classified within normal MC. Therefore, these findings provide empirical evidence, demonstrating the effectiveness of a structured motor skill curricular program implemented in PE focused on the result and not on the process.

Future studies should place special emphasis on research for this type of intervention with a medium- and long-term follow-up to see if these improvements in MC are maintained over time and study the process together with the result. Added to this, more studies are needed in other school settings (remembering that boys and girls spend an average of 5 h in schools) such as recess, the implementation of specific work schedules for motor skills adapted to ages and gender. The most important thing is for professionals to be aware of the importance of the development of MC in children, as these are the basis of their future development at the motor level.

## Figures and Tables

**Table 1:** MABC-2 test.

Test Dimension (Range)	Sub-Test
**Manual dexterity**	1st: Post coins (MD1)
	2nd: Threading beads (MD2)
	3rd: Drawing trail (MD3)
**Aiming and catching**	4th: Catching bean bag (AC1)
	5th: Throwing bean bag onto mat (AC2)
**Balance**	6th: One-leg balance (Bal1)
	7th: Walking heels raised (Bal2)
	8th: Jumping on mats (Bal3)
**Total test score (1–19)**	MD1 + MD2 + MD3 + AC1 + AC2 + Bal1 + Bal2 + Bal3
**Total percentile score (0.1–99.9)**	

**Table 2:** Objectives worked in each of the 6 sessions.

Session Number	Objectives	Tasks (Skills)
Session 1 “I explore my body”	Introduce manual dexterity, balance and global throwing and catching skills through games	“We play with the tweezers” (manual dexterity) “Balance chase game” (balance) “Do not fall!” (aiming and catching) “Manual golf” (aiming and catching) “The jumping kangaroos” (balance)
Session 2 “I develop my motor skills”	Improve fine motor and manual dexterity, jot down tasks, grasp and balance	“Wrap the giraffe” (manual dexterity) “Shooting into the tunnel” (aiming and catching) “Balance circuit” (balance)
Session 3 “The art of catching”	Develop manual dexterity with both hands and practice the tasks of catching and receiving various objects	“Chinese carriers” (manual dexterity) “Catch practice” (catching) “Catch and win” (catching) “Molded animals” (manual dexterity)
Session 4 “Sharpen your aim”	Improve fine motor skills in both hands. Develop aim and precision when throwing objects	“The coin catcher” (manual dexterity) “Aim for the bullseye” (aiming) “Double throw” (aiming and catching) “The labyrinth” (manual dexterity)
Session 5: “Circus tightrope walkers”	Work on manual dexterity and fine motor skills, develop static and dynamic balance	“Paste-stickers” (manual dexterity) “The stilts” (balance) “The rescue” (balance and aiming and catching) “The endless line” (balance) “To pick up!”
Session 6: “Motor circuits”	Remember through the motor circuit, tasks and games performed in previous sessions. Work with manual dexterity, aiming, grip and balancing	“The circuit” (manual dexterity; aiming and catching; balance) “Circuit 1” (manual dexterity; aiming and catching; balance) “Circuit 2” (manual dexterity; aiming and catching; balance)

**Table 3:** Results of MABC-2 test before intervention based on the type of group and gender.

		Total Sample		Control Group		Intervention Group	
		Mean	SD	Mean	SD	Mean	SD
Manual dexterity	Boys	6.27	2.69	4.00	0.00	4.20	2.04
	Girls	5.33	2.06	8.00	1.89	8.00	2.04
	Total	6.07	2.56	7.00	2.36	4.83	2.36
Aiming and catching	Boys	3.81	2.46	2.83	1.11	5.00	3.12
	Girls	2.33	0.51	2.00	0.00	3.00	0.00
	Total	3.50	2.26	2.62	1.02	4.66	2.93
Balance	Boys	3.00	2.46	2.50	1.97	3.60	2.95
	Girls	4.00	1.78	5.00	1.15	2.00	0.00
	Total	3.21	2.34	3.12	2.09	3.33	2.74
Total test score	Boys	2.45	1.65	2.50	1.88	2.40	1.42
	Girls	1.66	0.51	1.50	0.57	2.00	0.00
	Total	2.28	1.51	2.25	1.69	2.33	1.30
Total percentile score	Boys	1.58	2.14	1.80	2.36	1.32	1.94
	Girls	0.36	0.20	0.30	0.23	0.50	0.00
	Total	1.32	1.96	1.42	2.13	1.18	1.78

Note: SD = standard deviation.

**Table 4:** Results of MABC-2 test after intervention based on type of group and gender.

		Total Sample		Control Group		Intervention Group	
		Mean	SD	Mean	SD	Mean	SD
Manual dexterity	Boys	9.00	1.69	8.33	1.66	9.80	1.39
	Girls	7.33	4.13	6.00	4.61	10.00	0.00
	Total	8.64	2.42	7.75	2.72	9.83	1.26
Aiming and catching	Boys	6.36	2.80	4.66	2.22	8.40	1.95
	Girls	3.33	1.36	2.50	0.57	5.00	0.00
	Total	5.71	2.83	4.12	2.15	7.83	2.20
Balance	Boys	6.81	3.40	5.83	4.10	8.00	1.88
	Girls	9.33	2.73	8.00	2.30	12.00	0.00
	Total	7.35	3.39	6.37	3.79	8.66	2.30
Total test Score	Boys	6.36	2.83	4.66	2.60	8.40	1.42
	Girls	5.33	3.14	3.50	1.73	9.00	0.00
	Total	6.14	2.87	4.37	2.41	8.50	1.31
Total Percentile Score	Boys	18.69	17.42	8.60	9.41	30.80	17.36
	Girls	14.16	17.82	2.75	2.59	37.00	0.00
	Total	17.72	17.27	7.13	8.55	31.83	15.89

Note: SD = standard deviation.

## Data Availability

The data presented in this study are not available in accordance with Regulation (EU) of the European Parliament and of the Council 2016/679 of 27 April 2016 regarding the protection of natural persons with regard to the processing of personal data and the free circulation of these data (RGPD).

## References

[1] Bornstein M.H., Hendricks C. (2013). Screening for developmental disabilities in developing countries. Soc. Sci. Med..

[2] Hendrix C.G., Prins M.R., Dekkers H. (2014). Developmental coordination disorder and overweight and obesity in children: A systematic review. Obes. Rev..

[3] Lingam R., Jongmans M.J., Ellis M., Hunt L.P., Golding J., Emond A. (2012). Mental Health Difficulties in Children With Developmental Coordination Disorder. Pediatrics.

[4] Yu J., Sit C.H.P., Capio C.M., Burnett A., Ha A.S.C., Huang W.Y.J. (2016). Fundamental movement skills proficiency in children with developmental coordination disorder: Does physical self-concept matter?. Disabil. Rehabil..

[5] Clark J.E., Metcalfe J.S., Clark J.E., Humphrey J. (2002). The mountain of motor development: A metaphor. Motor Development: Research and Reviews.

[6] Cobley S., Abraham C., Baker J. (2008). Relative age effects on physical education attainment and school sport representation. Phys. Educ. Sport Pedagog..

[7] Almond L. (2014). Serious flaws in an FMS interpretation of physical literacy. Sci. Sports.

[8] Malina R.M., Bouchard C., Bar-Or O. (2004). Growth, Maturation, and Physical Activity.

[9] Bardid F., Deconinck F.J.A., Descamps S., Verhoeven L., De Pooter G., Lenoir M., D’Hondt E. (2013). The effectiveness of a fundamental motor skill intervention in pre-schoolers with motor problems depends on gender but not environmental context. Res. Dev. Disabil..

[10] Castro Zubizarreta A., Ezquerra Muñoz M.P., Argos González J. (2017). Fundamentos Teóricos de la Educación Infantil.

[11] Gallahue D.L., Ozmun J.C., Goodway J. (2012). Understanding Motor Development: Infants, Children, Adolescents, Adults.

[12] Stodden D.F., Goodway J.D., Langendorfer S.J., Roberton M.A., Rudisill M.E., Garcia C., Garcia L.E. (2008). A Developmental Perspective on the Role of Motor Skill Competence in Physical Activity: An Emergent Relationship. Quest.

[13] Fisher A., Reilly J.J., Kelly L.A., Montgomery C., Williamson A., Paton J.Y., Grant S. (2005). Fundamental Movement Skills and Habitual Physical Activity in Young Children. Med. Sci. Sport. Exerc..

[14] Lubans D.R., Morgan P.J., Cliff D.P., Barnett L.M., Okely A.D. (2010). Fundamental Movement Skills in Children and Adolescents. Sport. Med..

[15] Williams H.G., Pfeiffer K.A., O’Neill J.R., Dowda M., McIver K.L., Brown W.H., Pate R.R. (2008). Motor Skill Performance and Physical Activity in Preschool Children. Obesity.

[16] American Psychiatric Association (2013). Diagnostic and Statistical Manual of Mental Disorders.

[17] Roth K., Ruf K., Obinger M., Mauer S., Ahnert J., Schneider W., Graf C., Hebestreit H. (2010). Is there a secular decline in motor skills in preschool children?. Scand. J. Med. Sci. Sports.

[18] Beltrame T.S., Capistrano R., Alexandre J.M., Lisboa T., Andrade R.D., Felden É.P.G. (2017). Prevalência do Transtorno do Desenvolvimento da Coordenação em uma amostra de crianças brasileiras. Cad. Bras. Ter. Ocup..

[19] Pulzi W., Rodrigues G.M. (2015). Transtorno do Desenvolvimento da Coordenação: Uma Revisão de Literatura. Rev. Bras. Educ. Espec..

[20] Dos Santos V.A.P., Vieira J.L.L. (2013). Prevalência de desordem coordenativa desenvolvimental em crianças com 7 a 10 anos de idade. Rev. Bras. Cineantropometria Desempenho Hum..

[21] Wilson P.H., Ruddock S., Smits-Engelsman B., Polatajko H., Blank R. (2013). Understanding performance deficits in developmental coordination disorder: A meta-analysis of recent research. Dev. Med. Child Neurol..

[22] Amador-Ruiz S., Gutierrez D., Martínez-Vizcaíno V., Gulías-González R., Pardo-Guijarro M.J., Sánchez-López M. (2018). Motor Competence Levels and Prevalence of Developmental Coordination Disorder in Spanish Children: The MOVI-KIDS Study. J. Sch. Health.

[23] Wang T.-N., Tseng M.-H., Wilson B.N., Hu F.-C. (2009). Functional performance of children with developmental coordination disorder at home and at school. Dev. Med. Child Neurol..

[24] Deconinck F.J.A., Savelsbergh G.J.P., De Clercq D., Lenoir M. (2010). Balance problems during obstacle crossing in children with Developmental Coordination Disorder. Gait Posture.

[25] Bart O., Jarus T., Erez Y., Rosenberg L. (2011). How do young children with DCD participate and enjoy daily activities?. Res. Dev. Disabil..

[26] King-Dowling S., Missiuna C., Rodriguez M.C., Greenway M., Cairney J. (2015). Co-occurring motor, language and emotional–behavioral problems in children 3–6years of age. Hum. Mov. Sci..

[27] Magalhães L.C., Cardoso A.A., Missiuna C. (2011). Activities and participation in children with developmental coordination disorder: A systematic review. Res. Dev. Disabil..

[28] Prunty M.M., Barnett A.L., Wilmut K., Plumb M.S. (2014). An examination of writing pauses in the handwriting of children with Developmental Coordination Disorder. Res. Dev. Disabil..

[29] Rosa Neto F., Dos Santos A.P.M., Xavier R.F.C., Amaro K.N. (2010). A importância da avaliação motora em escolares: Análise da confiabilidade da escala de desenvolvimento motor. Rev. Bras. Cineantropometria Desempenho Hum..

[30] Pless M., Carlsson M., Sundelin C., Persson K. (2000). Effects of Group Motor Skill Intervention on Five- to Six-Year-Old Children with Developmental Coordination Disorder. Pediatr. Phys. Ther..

[31] Graupera J.L., Ruiz L.M. (2012). Batería de Evaluación del Movimiento Para Niños-2.

[32] Blank R., Smits-Engelsman B., Polatajko H., Wilson P. (2012). European Academy for Childhood Disability (EACD): Recommendations on the definition, diagnosis and intervention of developmental coordination disorder (long version). Dev. Med. Child Neurol..

[33] Schoemaker M.M., Smits-Engelsman B.C.M. (2015). Is Treating Motor Problems in DCD Just a Matter of Practice and More Practice?. Curr. Dev. Disord. Rep..

[34] Honrubia-Montesinos C., Gil-Madrona P., Losada-Puente L. (2021). Motor Development among Spanish Preschool Children. Children.

[35] Gao Z., Zeng N., Pope Z.C., Wang R., Yu F. (2019). Effects of exergaming on motor skill competence, perceived competence, and physical activity in preschool children. J. Sport Heal. Sci..

[36] Palmer K.K., Chinn K.M., Robinson L.E. (2019). The effect of the CHAMP intervention on fundamental motor skills and outdoor physical activity in preschoolers. J. Sport Health Sci..

[37] Hillier S. (2007). Intervention for Children with Developmental Coordination Disorder: A Systematic Review. Internet J. Allied Health Sci. Pract..

[38] Smits-Engelsman B.C.M., Blank R., Van Der Kaay A.-C., Mosterd-Van Der Meijs R., Vlugt-Van Den Brand E., Polatajko H.J., Wilson P.H. (2013). Efficacy of interventions to improve motor performance in children with developmental coordination disorder: A combined systematic review and meta-analysis. Dev. Med. Child Neurol..

[39] Yu J.J., Burnett A.F., Sit C.H. (2018). Motor Skill Interventions in Children With Developmental Coordination Disorder: A Systematic Review and Meta-Analysis. Arch. Phys. Med. Rehabil..

[40] Caçola P., Romero M., Ibana M., Chuang J. (2016). Effects of two distinct group motor skill interventions in psychological and motor skills of children with Developmental Coordination Disorder: A pilot study. Disabil. Health J..

[41] Hung W.W.Y., Pang M.Y.C. (2010). Effects of group-based versus individual-based exercise training on motor performance in children with developmental coordination disorder: A randomized controlled pilot study. J. Rehabil. Med..

[42] Dunford C. (2011). Goal-Orientated Group Intervention for Children with Developmental Coordination Disorder. Phys. Occup. Ther. Pediatr..

[43] Ato M., López J.J., Benavente A. (2013). Un sistema de clasificación de los diseños de investigación en psicología. An. Psicol..

[44] Henderson S.E., Sudgen D.A., Barnett A. (2007). Movement Assessment Battery for Children-2.

[45] Schoemaker M.M., Niemeijer A.S., Flapper B.C.T., Smits-Engelsman B.C.M. (2012). Validity and reliability of the Movement Assessment Battery for Children-2 Checklist for children with and without motor impairments. Dev. Med. Child Neurol..

[46] Valentini N.C., Ramalho M.H., Oliveira M.A. (2014). Movement assessment battery for children-2: Translation, reliability, and validity for Brazilian children. Res. Dev. Disabil..

[47] Serbetar I., Loftesnes J.M., Mamen A. (2019). Reliability and Structural Validity of the Movement Assessment Battery for Children-2 in Croatian Preschool Children. Sports.

[48] Ministerio de Educación y Ciencia (2007). Real Decreto 1630/2006, de 29 de Diciembre, por el que se Establecen las Enseñanzas Mínimas del Segundo ciclo de Educación Infantil.

[49] Logan S.W., Robinson L.E., Wilson A.E., Lucas W.A. (2012). Getting the fundamentals of movement: A meta-analysis of the effectiveness of motor skill interventions in children. Child. Care. Health Dev..

[50] Thornton A., Licari M., Reid S., Armstrong J., Fallows R., Elliott C. (2016). Cognitive Orientation to (Daily) Occupational Performance intervention leads to improvements in impairments, activity and participation in children with Developmental Coordination Disorder. Disabil. Rehabil..

[51] Au M.K., Chan W.M., Lee L., Chen T.M.K., Chau R.M.W., Pang M.Y.C. (2014). Core stability exercise is as effective as task-oriented motor training in improving motor proficiency in children with developmental coordination disorder: A randomized controlled pilot study. Clin. Rehabil..

[52] Ferguson G.D., Jelsma D., Jelsma J., Smits-Engelsman B.C.M. (2013). The efficacy of two task-orientated interventions for children with Developmental Coordination Disorder: Neuromotor Task Training and Nintendo Wii Fit training. Res. Dev. Disabil..

[53] Noordstar J.J., van der Net J., Voerman L., Helders P.J.M., Jongmans M.J. (2017). The effect of an integrated perceived competence and motor intervention in children with developmental coordination disorder. Res. Dev. Disabil..

[54] Wilson P.H., Adams I.L.J., Caeyenberghs K., Thomas P., Smits-Engelsman B., Steenbergen B. (2016). Motor imagery training enhances motor skill in children with DCD: A replication study. Res. Dev. Disabil..

[55] Jongmans M.J., Linthorst-Bakker E., Westenberg Y., Smits-Engelsman B.C.M. (2003). Use of a task-oriented self-instruction method to support children in primary school with poor handwriting quality and speed. Hum. Mov. Sci..

[56] Jongmans M.J., Smits-Engelsman B.C.M., Schoemaker M.M. (2003). Consequences of Comorbidity of Developmental Coordination Disorders and Learning Disabilities for Severity and Pattern of Perceptual—Motor Dysfunction. J. Learn. Disabil..

[57] Farhat F., Hsairi I., Baati H., Smits-Engelsman B.C.M., Masmoudi K., Mchirgui R., Triki C., Moalla W. (2016). The effect of a motor skills training program in the improvement of practiced and non-practiced tasks performance in children with developmental coordination disorder (DCD). Hum. Mov. Sci..

[58] Smits-Engelsman B., Vinçon S., Blank R., Quadrado V.H., Polatajko H., Wilson P.H. (2018). Evaluating the evidence for motor-based interventions in developmental coordination disorder: A systematic review and meta-analysis. Res. Dev. Disabil..

[59] Wood G., Miles C.A.L., Coyles G., Alizadehkhaiyat O., Vine S.J., Vickers J.N., Wilson M.R. (2017). A randomized controlled trial of a group-based gaze training intervention for children with Developmental Coordination Disorder. PLoS ONE.

[60] Niemeijer A.S., Smits-Engelsman B.C.M., Schoemaker M.M. (2007). Neuromotor task training for children with developmental coordination disorder: A controlled trial. Dev. Med. Child Neurol..

